# Comprehensive Understanding of Silicon-Nanowire Field-Effect Transistor Impedimetric Readout for Biomolecular Sensing

**DOI:** 10.3390/mi12010039

**Published:** 2020-12-31

**Authors:** Abhiroop Bhattacharjee, Thanh Chien Nguyen, Vivek Pachauri, Sven Ingebrandt, Xuan Thang Vu

**Affiliations:** 1 Institute of Materials in Electrical Engineering 1, RWTH Aachen University, Sommerfeldstraße 24, 52074 Aachen, Germany; abhiroop1006@gmail.com (A.B.); pachauri@iwe1.rwth-aachen.de (V.P.); ingebrandt@iwe1.rwth-aachen.de (S.I.); 2 Department of Informatics and Microsystem Technology, University of Applied Sciences Kaiserslautern, Amerikastrasse 1, 66482 Zweibrücken, Germany; ntchien82@gmail.com; 3 Department of Electrical and Electronics Engineering, Birla Institute of Technology and Science Pilani (BITS Pilani), Pilani 333031, India

**Keywords:** silicon-nanowire field-effect transistor, impedimetric readout, biosensors, simulation program with integrated circuit emphasis (SPICE), transistor transfer function

## Abstract

Impedance sensing with silicon nanowire field-effect transistors (SiNW-FETs) shows considerable potential for label-free detection of biomolecules. With this technique, it might be possible to overcome the Debye-screening limitation, a major problem of the classical potentiometric readout. We employed an electronic circuit model in Simulation Program with Integrated Circuit Emphasis (SPICE) for SiNW-FETs to perform impedimetric measurements through SPICE simulations and quantitatively evaluate influences of various device parameters to the transfer function of the devices. Furthermore, we investigated how biomolecule binding to the surface of SiNW-FETs is influencing the impedance spectra. Based on mathematical analysis and simulation results, we proposed methods that could improve the impedimetric readout of SiNW-FET biosensors and make it more explicable.

## 1. Introduction

Since the introduction of the first ion-sensitive field-effect transistor (ISFET) by Piet Bergveld, researchers have been striving to use ISFETs effectively as biosensors [1]. With advancements in the development of nanomaterials and nanoscale fabrication, a new class of biosensors based on SiNW-FET have emerged with better sensitivity and unprecedented functionality [2,3]. Being sub-micrometer sized in at least one dimension, SiNW-FET based biosensors possess very high surface-volume ratios [3,4,5]. Biosensors based on SiNW-FETs, being highly sensitive, are suitable for label-free detection of biomolecules and are characterized by large dynamic ranges as well as fast responses [2,3,6,7,8,9]. They can be used for various applications such as immuno-assays [4,5], DNA hybridization [10], and detection of bacteria or viruses [11].

The classical readout principle of SiNW-FET biosensors is based on a potentiometric method that involves a change in surface potential at the solid-liquid interface of SiNW-FETs [4,5,12,13,14]. Unlike a metal-oxide semiconductor field-effect transistor (MOSFET), which employs a metal or polysilicon as gate electrode material, a SiNW-FET biosensor employs a system of a reference electrode and an electrolyte solution as a gate electrode contact. The gate voltage is applied thought the reference electrode to set the operating point of the device. The potential dropped between the reference electrode and the source of the SiNW-FET is the sum of many interfacial potentials [2,15,16]. In biosensing experiments, the surface potential at the solid-liquid interface is varied by the binding of charged biomolecules to the sensor surface [4,5,12,13,14]. The change of the surface potential acts as an additional gate voltage leading to a change in the threshold voltage of the device. The biomolecules of interest are detected by measuring the change in the threshold voltage [17,18,19] or the change in the drain-source current (*I_DS_*) [4,5,10] of the SiNW-FET device upon the binding to specific receptor molecules at the gate surface. Their concentration can be calculated accordingly by applying the respective adsorption models [20]. The sensitivity and selectivity of the SiNW-FET sensors can be improved by selecting a proper surface functionalization for the immobilization of the capture molecules [21]. The limits of detection of SiNW-FETs are typically in the range of femto molar concentration of biomolecules [5]. Close to the limit of detection, the change in signal is small in the range of a few mV in threshold voltage or a few nanoamperes in the drain-source current. This mainly depends on the dimension of the SiNW-FET and the quality of surface functionalization. To measure such small signal changes high-resolution and low-noise amplifiers are required. In addition, it is well known that the potentiometric readout method is limited by the Debye-screening effect [22,23,24,25], which also undermines the application of the SiNW-FETs as point-of-care biosensor working in undiluted biological samples or even in whole blood.

To address these issues, in earlier works we introduced a novel approach based on the frequency response of SiNW-FETs as an alternative readout method [26,27]. This technique was earlier employed for the detection of DNA, protein, and adhesion of individual cells using microscale ISFET devices [27,28,29,30,31,32]. In this method, the transistor is set to an operating point—normally at maximum transconductance—and a small sinusoidal signal, 5−10 mV, is added to its gate electrode and the signal transfer function of the device and its readout circuit is measured. The response of the transistor at different frequencies is then recorded, and an impedance spectrum is analyzed similar to classical electrochemical impedance spectroscopy. Biomolecular binding events on the gate surface cause a change in the frequency response, which is mostly prevalent at the low-pass characteristics. Through this impedimetric sensing technique, Schwartz et al. [27] demonstrated that it was possible to detect DNA hybridization in high ionic strength buffer solutions close to physiological concentrations using SiNW-FETs at frequency of 100 kHz and above, thereby overcoming the classical Debye-screening limitation of potentiometric sensing. It was also possible to sense tiny charged as well as even uncharged molecules, which were otherwise indistinguishable in case of potentiometric sensing [8,33]. It was also demonstrated that in comparison with standard potentiometric measurements, impedimetric sensing yielded a significant improvement in the biosensor performance, including the limit of detection and dynamic range [8]. Even though, many experimental studies showed successful detection of different biomolecules with this technique, a comprehensive understanding is still missing.

In previous studies [26], we introduced and validated a SPICE behavioral macro model and developed an electrically equivalent circuit for SiNW-FETs to understand the effect of device geometries such as drain and source parasitic capacitances as well as the effect of conductivity and pH of the electrolyte solution on the transfer function of the system. The previous results helped us in designing a readout system for the SiNW-FET biosensor arrays [34]. In this paper, we employed the existing SPICE model and developed an equivalent circuit of the SiNW-FET sensors with biomolecules on the gate to explore a mathematical basis for the sensor response in the label-free detection of biomolecules. We proposed a transistor-transfer function (TTF) for the SiNW-FETs taking into account the various parameters of the SiNW-FET model [26,34] and the parasitic capacitances due to the source *C_ps_* and the drain *C_pd_* areas of the SiNW-FET [26]. Thereafter, we studied the effect of biomolecular binding at the SiNW-FET by an appropriate simulation in SPICE and then quantify our observations. Furthermore, we proposed future directions to make the biomolecular detection with the TTF method for SiNW-FETs more explicable.

## 2. Electronic Circuit Model for SiNW-FET and Readout Apparatus

**Figure 1:**
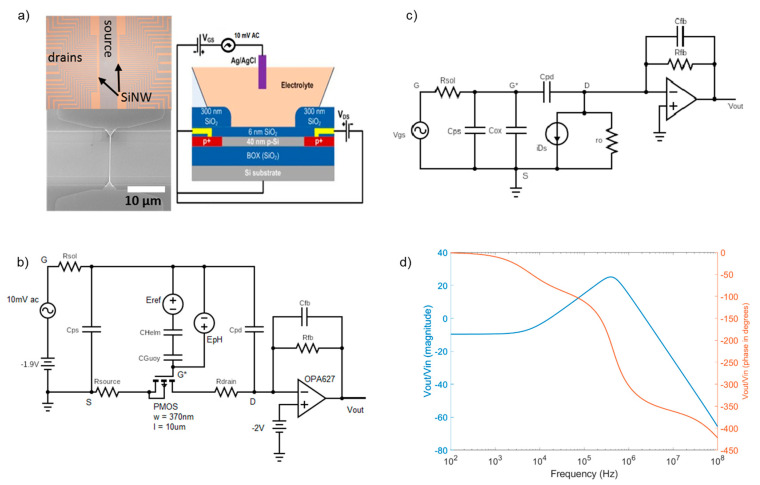
(**a**) Optical image, SEM image and cross-section view of the SiNW-FET arrays used in the simulation model, (**b**) Electrically equivalent circuit for the SiNW-FET including a transistor, parameters of source and drain contacts and of the electrolyte; (**c**) *ac* small-signal model for the SiNW ISFET with the readout operational amplifier, (**d**) magnitude and phase plots for the TTF in presence of the parasitic capacitances *C_ps_* and *C_pd._*

The top-down fabrication protocol of SiNW-FET arrays on 4-inch Silicon-on-Insulator (SOI) wafers (SOITEC, Bernin, France) having a common source electrode design, was previously described [19]. We combined wafer-scale nanoimprint lithography and wet anisotropic etching of silicon with tetramethylammonium hydroxide (TMAH) to define nanowires and contact lines in one lithography step. Boron ions were implanted on the contact lines to reduce the serial resistances, while retaining the high charge carrier mobility inside the SiNWs. Chips were passivated by a 300 nm thick layer of SiO_2_ by low pressure chemical vapor deposition (LPCVD). A thin layer of 6 nm SiO_2_ was dry thermally grown on the SiNWs, which serves as the gate insulator. The SiNW-FET used in this work had a layout of 28 × 2 channels and had a channel length of 10 µm and a channel width of 370 nm, respectively. The devices were working as long-channel p-type transistors [19]. An optical image of the chip, an SEM image of the SiNW-FET and its cross-sectional view are presented in Figure 1a.

A behavioral SPICE simulation model (BSIMv3 Level 7) for the SiNW-FET was earlier adopted for impedimetric measurements [26,34]. Various BSIMv3 simulation parameters for the SiNW-FET model are listed in Table 1. The major parameters, which were identified from the SiNW-FET chips in previous studies [19,27], were the width (*width*), the length (*length*), the thickness (*t_si_*) of the SiNW-FET, the thickness of the gate SiO_2_ layer (*t_ox_*), the carrier mobility (*U*_0_*),* doping concentration near channel interface (*N_ch_*) and the threshold voltage (*V_th_*_0_). Previous studies validated that the model level 7 accurately mimics the SiNW-FET characteristics [19,26,34].

Figure 1b depicts the scheme for the electrically equivalent circuit for the SiNW-FET sensor, which includes two stages. First, the SiNW-FET is modelled as an equivalent long channel p-type MOSFET with the electrolyte-oxide interface including capacitance and resistance of the drain *C_pd_, R_drain_* and of the source *C_ps_*, *R_source_*. The solid-liquid interface at the gate is modelled by Helmholtz and Gouy-Chapman capacitances in series [26]. *E_pH_* depicts the potential variation at the solid-liquid interface due to pH of the electrolyte. Electrolyte resistance *R_sol_* presents the conductivity of electrolyte. A detailed description of this model and all its parameters can be found in a previous publication [26]. Second, the readout circuit is based on the transimpedance amplifier principle consisting of a high bandwidth operational amplifier (OPA627, Texas Instruments, Dallas, TX, USA), and its feedback resistor *R_fb_* and capacitor *C_fb_*.

An electrically equivalent circuit for the *ac* small-signal model of the SiNW-FET and the transimpedance amplifier is shown in Figure 1c Transfer function of the sensor is the ratio between the output voltage after the transimpedance amplifier (*v_out_*) and the input voltage at the gate of the sensor (*v_in_*). The transfer function spectrum of the sensors simulated with the above parameters for the frequency ranging from 100 Hz to 100 MHz is presented in Figure 1d. A detailed explanation of the SiNW-FET transfer function and its mathematical model will be closer examined in Section 3.

## 3. Mathematical Formulation of Transfer Function

We begin the analysis assuming *C_pd_* = 0 F. For the circuit shown in Figure 1b, the frequency response of the output voltage *v_out_* can be derived.

(1) $$v_{out}=\frac{g_{m}R_{fb}v_{gs}}{\left(1+\frac{s}{\frac{1}{R_{fb}C_{fb}}}\right)\left(1+\frac{s}{\frac{1}{R_{sol}\left(C_{ps}+C_{ox}\right)}}\right)}$$

Here, *s = jω*; *j* is the imaginary number and *ω* is angular frequency. The output voltage represents a 2-pole system with a *dc* gain defined by *g_m_R_fb_* and the two poles being *p*_1_ = $\frac{1}{2\pi R_{fb}C_{fb}}$, which represents the transimpedance amplifier, and *p*_2_ = $\frac{1}{2\pi R_{sol}\left(C_{ps}+C_{ox}\right)}$, which represents the SiNW-FET. In this SiNW-FET model *C_ps_* ≫ *C_ox_*, so the effect of *C_ox_* is neglected and the second pole is $\frac{1}{2\pi R_{sol}C_{ps}}$. The magnitude response of this system shall be identical to that of a low-pass filter [26]. However, an occurrence of a peak in the magnitude spectrum of *v_out_* without any change in the *dc* gain is observed as it can be seen in Figure 1d around 600 kHz. This is due to the effect of the capacitance *C_pd_*, as in practice *C_pd_* is a finite value. So, a finite *C_pd_* introduces a zero frequency in the spectrum apart from the two poles discussed earlier. The value of the zero frequency due to *C_pd_* can be qualitatively estimated using following approximations: The drain- source current *i_ds_* of the SiNW-FET, as represented in Figure 1c, can be considered as a resistor with resistance equal to $\frac{1}{g_{m}}$ as the pMOS can be approximately considered to be in a source-follower configuration, which makes v_G*S_ = v_DS_. Thus, the zero frequency would be a consequence of the nanowire resistance $\frac{1}{g_{m}}$ and the drain capacitance *C_pd_*. Corresponding to this zero frequency, instead of a rise in the phase of the spectrum, a drop in the phase is observed as it can be seen in Figure 1d. Therefore, the factor to be multiplied to the numerator of the transfer function in Equation (1), corresponding to the zero frequency (*z*), must be of the form $(1-\frac{s}{z}$) rather than $(1+\frac{s}{z}$) [35]. The second pole also gets modified in the presence of the drain capacitance as *p*_2*_ = $\frac{1}{2\pi R_{sol}\left(C_{ps}+C_{ox}+C_{pd}\right)}$. In the SiNW-FET model, *C_ps_* ≫ *C_ox_* and *C_pd_* ≫ *C_ox_*, so the second pole is approximately equal to $\frac{1}{2\pi R_{sol}\left(C_{ps}+C_{pd}\right)}$. The Equation (1) is modified with the zero frequency $\frac{g_{m}}{2\pi C_{pd}}$ and the modified second pole is represented as

(2) $$v_{out}=\frac{g_{m}R_{fb}v_{gs}\left(1-\frac{sC_{pd}}{g_{m}}\right)}{\left(1+sR_{fb}C_{fb}\right)\left(1+sR_{sol}\left(C_{ps}+C_{pd}\right)\right)}$$

with the first pole *p*_1_ = $\frac{1}{2\pi R_{fb}C_{fb}}$, second pole *p*_2_ = $\frac{1}{2\pi R_{sol}\left(C_{ps}+C_{pd}\right)}$, and the zero frequency *z*_1_ = $\frac{g_{m}}{2\pi C_{pd}}$.

## 4. Investigation of the Effect of the Device Geometries and of the Electrolyte Solution on the Transfer Function

The initially selected values of all parameters for our studies are shown in Table 2. All values were adapted from the SiNW-FET presented in our previous publications [26,34]. Unless otherwise stated in the text, all the simulation results are corresponding to these parameters.

**Figure 2:**
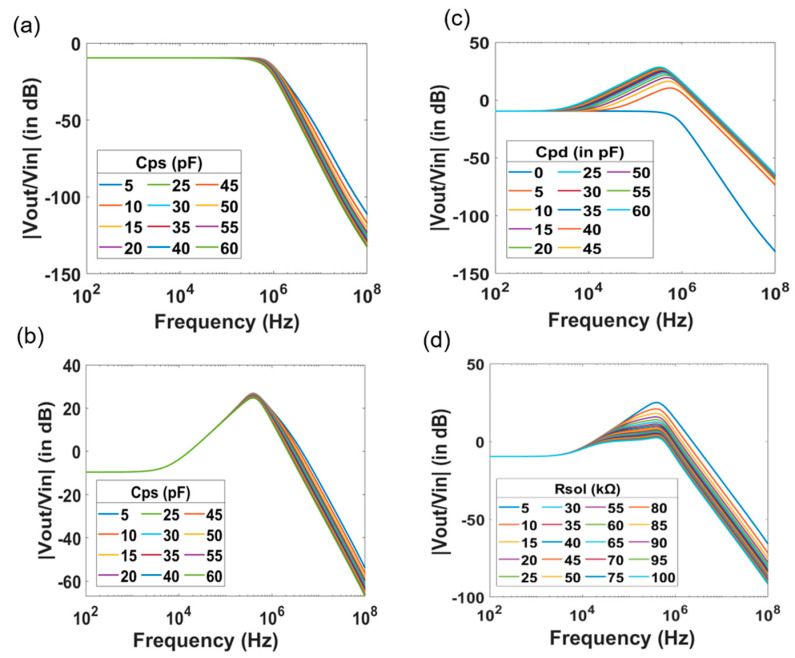
Effect of drain and source capacitances of the SiNW-FET as well as electrolyte parameters to the frequency spectrum. TTF spectrum of the SiNW-FET with varying source capacitance (*C_ps_*) for two scenario of drain capacitance with *C_pd_* = 0 fF (**a**) and *C_pd_* = 33 fF (**b**). (**c**) Effect the drain capacitance (*C_pd_*) while the source capacitance is equal to 50 fF. As *C_pd_* is introduced, a peak appears in the TTF spectra. An increase in *C_pd_* leads to a change in the amplitude and in the position of the peak. (**d**) Effect of conductivity of electrolyte on the TTF spectra. The peak amplitude decreases with the decrease of electrolyte concentration.

When *C_pd_* is set to zero, *z*_1_ is no longer present in the transfer function of the system. In this case, the spectrum is expected to be identical to that of a low pass filter having two poles. As *C_ps_* increases, *p*_2_ shifts towards lower frequencies. The simulation results in which *C_ps_* varied from 5 pF to 60 pF in steps of 5 pF are shown in Figure 2a.

In case *C_pd_* has a finite value, there are peaks in the spectrum. As the value of *C_pd_* increases, the peak increases in amplitude and shifts towards lower frequencies. Simulation results for a fix value of *C_pd_* = 33 pF and a stepwise variation of *C_ps_* from 5 pF to 60 pF in steps of 5 pF are presented in Figure 2b. As *C_ps_* increases, *p*_2_ gradually shifts to the vicinity of *z*_1_. At a particular instant, the effect of the zero frequency gets nullified by the pole due to *C_ps_*. Consequently, the effect of *p*_1_ only prevails, and the frequency response of the system becomes identical to that of a low pass filter with a single pole as shown in Figure 2c. Clearly, the simulation results are in agreement with the proposed transfer function. Figure 2d depicts the effect of varying *R_sol_* from 5 kΩ to 100 kΩ in steps of 5 kΩ. Increasing *R_sol_* implies shifting the pole *p*_2_ to lower frequencies closer to z_1_, which in turn causes the peak amplitudes to decrease in their amplitude values.

Furthermore, change in the thickness of the gate oxide *t_ox_* results in a change in *C_ox_*, thereby in a change of *g_m_*. Since, the *dc* gain of the system is *g_m_R_fb_*, the change in oxide thickness results in a *dc* shift of the magnitude response of the transfer function. As *C_ox_* = *A_ox_t_ox_* and *g_m_* ∝ $\sqrt{C_{ox}}$, the value of *g_m_* is inversely proportional to the square root of *t_ox_*, *g_m_* ∝ $\frac{1}{\sqrt{t_{ox}}}.$ This implies that for a two-fold change in *t_ox_*, there is a 6 dB shift in the *dc* gain of the transfer function.

**Figure 3:**
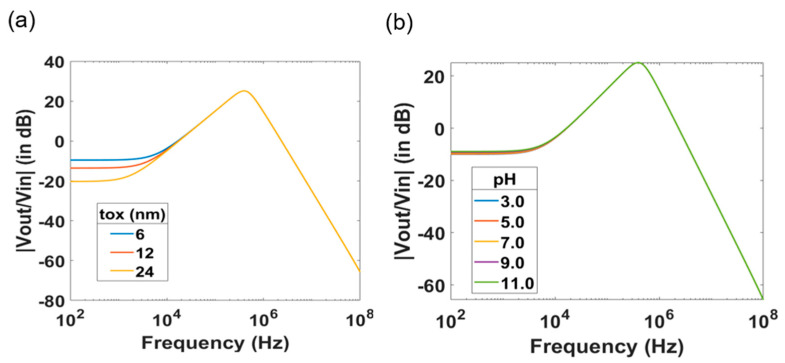
(**a**) Magnitude spectrum of the SiNW-FET sensor with *t_ox_* varying as 6 nm, 12 nm and 24 nm. The effect of the oxide thickness causes a change in the TTF spectrum at low frequencies. (**b**) Magnitude spectrum with pH of electrolyte varying from 3.0 to 11.0.

For different values of thicknesses of the gate oxide *t_ox_*, there is an initial *dc* shift in the magnitude between the curves. Then, the TTF spectrum merges at higher frequencies resulting in a single peak as shown in Figure 3a. This observation validates the expression proposed for *v_out_*. Thus, any change in the oxide thickness or gate capacitance translates into a shift in the *dc* gain of the transfer function. Similar to the case of variation in the gate oxide thickness, there is an initial *dc* shift due to the change of the surface potential caused by protonation and deprotonation of the gate oxide surface, as the pH value of the electrolyte changes. Figure 3b presents data obtained with different pH values ranging from 3 to 11 in steps of 2. The curves appear to merge at higher frequencies, resulting in a single peak. This is a consequence of the dependence of *g_m_* on the pH of the electrolyte. Nevertheless, this *dc* shift is not as prominent as in the case of varying *t_ox_*.

**Figure 4:**
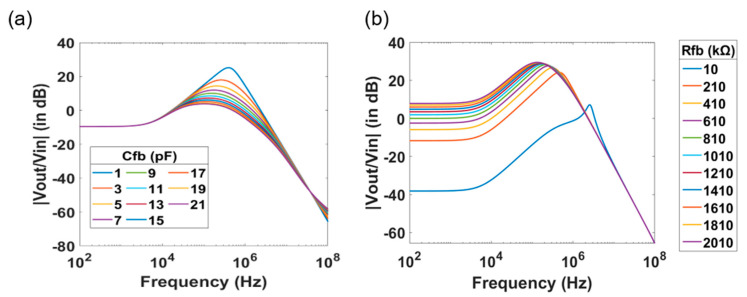
Magnitude spectrum with varying feedback parameters of the readout transimpedance amplifier. Change of the feedback capacitance (**a**) and feedback resistance (**b**) cause change in the amplitude and the position of the peak in the TTF spectra of SiNW-FET sensors.

After the influence of the SiNW-FET parameters and the effect of the electrolyte solution in terms of pH value and conductivity to the transfer function of the system is investigated. We further studied the effect of the readout circuit parameters by varying the feedback resistance *R_fb_* and the feedback capacitance *C_fb_*, while keeping all other parameters at a constant value. Simulated data are presented in Figure 4. Increasing *C_fb_* leads to a decrease of the peak amplitude and a shift of *p*_1_ to lower frequencies. Figure 4a represents the data with increasing *C_fb_* from 1 pF to 21 pF in steps of 2 pF. Similarly, increase in *R_fb_* also shifts *p*_1_ to lower frequencies. Unlike the case of increasing *C_fb_* or *R_sol_*, where there was a reduction in the peak amplitude without a *dc* shift, there is an increase in *dc* gain with increasing resistance, owing to the presence of *R_fb_* as a factor in the *dc* gain as shown in Figure 4b.

## 5. Impedimetric Detection of Biomolecules Using SiNW

**Figure 5:**
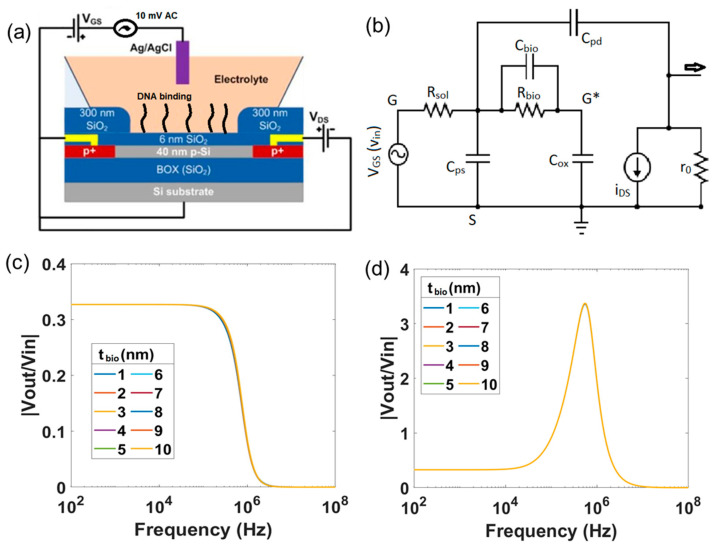
(**a**) Schematic of the DNA detection with a SiNW-FET considering the biomolecules only at the gate, while neglecting the interaction of the biomolecules with the drain and the source. (**b**) Electrically equivalent circuit for this measurement including the additional *RC* circuit elements caused by biomolecular layer on the gate. Magnitude spectrum of SiNW-FET with *t_bio_* varying from 1 to 10 nm in case *C_pd_* = 0 fF (**c**) and *C_pd_* = 33 fF (**d**), while other parameters stay constant.

Earlier studies considered the input impedance of the biomolecular layer to be an *RC* network consisting of a resistor *R_bio_* and a capacitor *C_bio_* connected in parallel and placed in series with the gate capacitor *C_ox_* [27,30,36,37]. A schematic of the SiNW-FET sensor for DNA hybridization detection and the corresponding electronically equivalent circuit (*ac* small-signal model) is shown in Figure 5a,b, respectively. Here, we introduce three new parameters: *R_bio_* is the electrical resistance of the biomolecular layer and is set to 5 MΩ, *t_bio_* is the thickness of the biomolecular layer and *κ_bio_* is its dielectric constant [28,38]. The capacitance of the biomolecular layer, *C_bio_*, is deduced from the surface area of the SiNW-FET, the thickness of the biomolecular layer, and its dielectric constant.

The simulation results with varying thickness of the biomolecular layer for both scenarios of *C_pd_* being equal to zero and *C_pd_* being finitely large are presented in Figure 5c,d, respectively. When the parasitic capacitance *C_pd_* is equal to zero, a pole *p*_3_, and a zero *z*_2_ are introduced in the spectrum of *v_out_*. The pole and zero frequencies can be calculated to

(3) $$p_{3}\;=\;\frac{1}{2\pi R_{bio}\left(C_{bio}+C_{pd}+C_{ox}\right)},\;z_{2}\;=\;\frac{1}{2\pi R_{bio}C_{bio}}$$

As it can be seen, there is only a small change in the impedance spectra upon a large change in the biomolecular layer. However, the presence of *z*_1_ in case *C_pd_* is finitely large masks the effect of this pole and zero. Hence, distinct curves in the spectrum for different molecular immobilization cannot be clearly observed (Figure 5d). Furthermore, we also do not observe any *dc* shift i.e., the *dc* gain is still equal to *g_m_R_fb_*.

**Figure 6:**
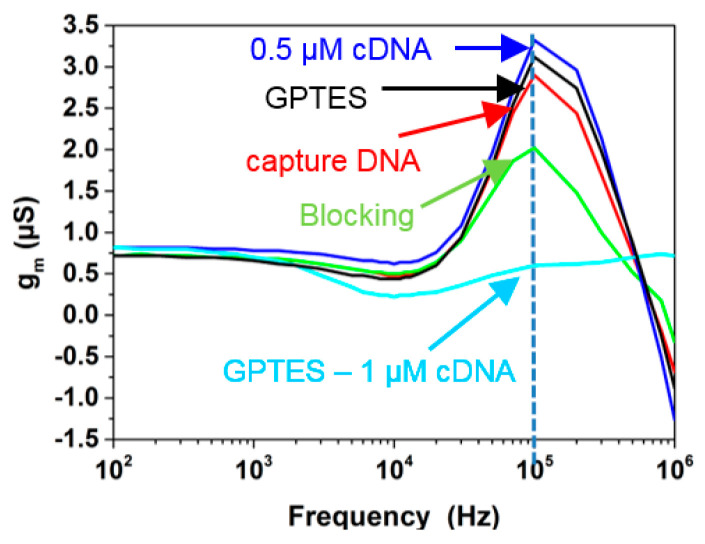
Magnitude spectrum of transconductance of a SiNW-FET for various biomolecular immobilizations [27]. The image has been reproduced with permission from [27].

However, in one of our previous studies [27], the transfer function of a SiNW-FET was measured in 0.001× PBS solution after each experimental step of silanization with (3-Glycidyloxypropyl)trimethoxysilan (GPTES), capture DNA immobilization, blocking with 1% BSA, and hybridization with 0.5 μM and 1 μM cDNA showed a different behavior, Figure 6. One can observe a strong change in the pole *p*_3_ in each experimental steps. Unlike the results of the simulations in Figure 5c,d, the experiments showed significantly different peaks for each experimental steps. From the calculated transfer function and simulation results in this study, the peak can be affected if *C_pd_* is altered, resulting in the variation of *z*_1_. But according to the circuit model described in Figure 5a,b, *C_pd_* remained constant during the simulations. Hence, it is clear that the experimented transfer function was not only affected by the biomolecular layer that bound to the SiNW-FET surface, but also by changes of other parameters like the *C_ps_*, *C_pd_* of the SiNW-FET or *R_sol_*.

**Figure 7:**
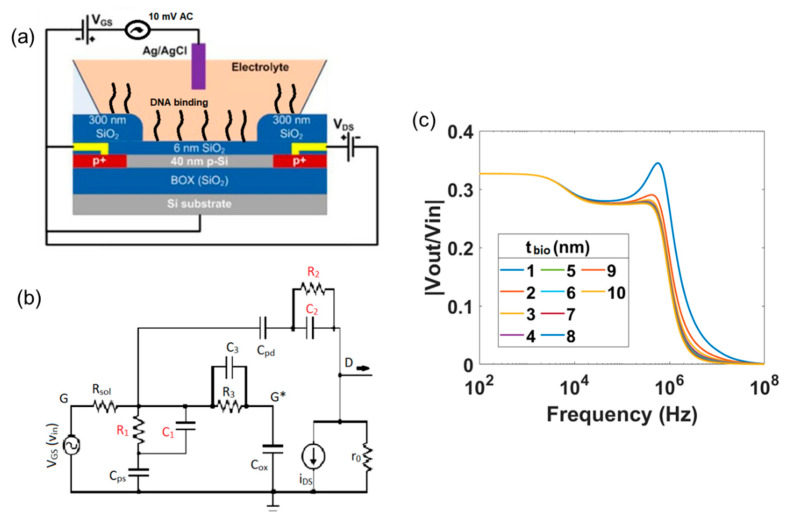
(**a**) Schematic of the DNA detection with a SiNW-FET taking the attachment of a biomolecular layer to the contact lines in to account as well. (**b**) Electrically equivalent circuit for this measurement including the additional *RC* circuit elements in red. (**c**) Magnitude spectrum with *t_bio_* varying from 1 to 10 nm showing much stronger changes in the spectrum.

So far, we have overlooked the interaction of the biomolecule layer with the source and drain contacts. However, based on the experimental method as it is described in previous studies [13,27], we cannot rule out the fact that these biomolecules would also bind to the drain and source contacts with the same manner as to the SiNW-FET surface. The schematic of the SiNW-FET including the binding of biomolecules to the gate as well as to the source and drain contacts is presented in Figure 7a. Hence, additionally to the *RC* network of the biomolecules on the gate, a similar *RC* network of the biomolecules is expected to be in series with *C_ps_* and *C_pd_*, respectively. We qualitatively analyzed the possible repercussions on consideration of this phenomenon. The modified electronically equivalent circuit is shown in Figure 7b and the simulation results are shown in Figure 7c for a thickness variation of *t_bio_* in the range 1–10 nm.

Qualitatively, the effective parasitic capacitances at the drain and source contacts would decrease with the intervention of the biomolecule layer as both the parasitic capacitance of source/drain contacts and the *RC* network model of the biomolecule layer are combined in series. As a result, a shift in the peak amplitude and the pole *p*_2_ of the transfer function is expected. With a decrease in *C_bio_*, the peak amplitude decreases and the pole *p*_2_ shifts towards higher frequencies. The pole *p*_3_ and zero *z*_2_ still prevail and solely depend on the *RC* network of biomolecular layer in series with *C_ox_*. The simulated results, which include the binding of the biomolecules to the source and drain contacts, represent the experimental data of Figure 6. This clearly indicates that the TTF of the SiNW-FET was affected both by the binding of the biomolecules on the gate as well as to the source and drain contact surfaces.

To overcome the effect of the drain contact capacitance or, in other words, to minimize the effect of parasitic capacitance of drain and source contacts caused by the binding of biomolecules, we implemented a microfluidic structure on top the SiNW-FET sensor and confined the binding of biomolecules within the microfluidic structure. The effect of the parasitic capacitances (*C_ps_* and *C_pd_*) can be minimized, whereas the interaction of the biomolecules at the gate of the SiNW-FET becomes the major effect. However, a small area of the drain and source contacts is still affected by the binding of biomolecules due to the immobilization method of the capture molecules [27]. To estimate *R_bio_*, a parameter λ called resistance per unit area in a unit of kΩ/m^2^ is defined. This resistance is influenced by the biomolecules on the source contact *R*_1_, on the drain contact *R*_2_ as well as on the gate *R*_3_. It can be calculated accordingly.

*R*_1_ = *R*_*bio (drain)*_ = *λ* * *(area of contact with drain)*

*R*_2_ = *R*_*bio (source)*_ = *λ* * *(area of contact with source)*

*R*_3_ = *R_bio_*_(*gate*)_ = *λ* * *A_ox_*

The capacitance per unit area in F/m^2^ of the binding biomolecules is defined as γ =$\frac{\epsilon_{bio}}{t_{bio}}$ and the related capacitances can be calculated as well.

*C*_1_ = *C*_*bio (drain)*_ = *γ* * *(area of contact with drain)*

*C*_2_ = *C*_*bio (source)*_ = *γ* * *(area of contact with source)*

*C*_3_ = *C*_*bio (gate)*_ = *γ* * *A*_*ox*_

**Figure 8:**
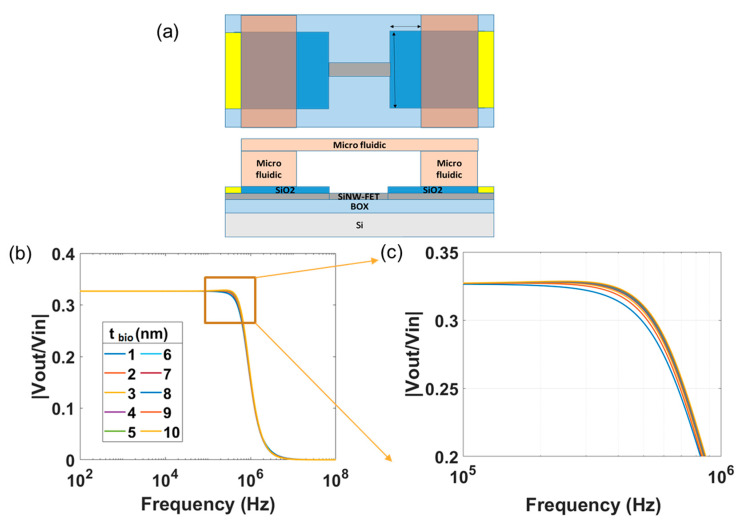
Magnitude spectrum with *t_bio_* varying from 1–10 nm, when we assume only binding of biomolecules inside a microfluidic structure. (**a**) Only small portions of each of the source and drain contact lines (1 μm × 10 μm) are exposed to biomolecules, which minimizes the effect of the parasitic capacitances. (**b**,**c**) Change in the impedance spectra in this case is mainly caused by the binding of the biomolecules on the SiNW-FET gate.

Simulation results with fixed source and drain contact areas of 1 μm × 10 μm inside a microfluidic and with the *t_bio_* varying in the range 1–10 nm are shown in Figure 8. The source and drain contact parasitic capacitances are computed as follows:

(4) $$C_{ps}=C_{pd}=\frac{A_{ox}\varepsilon_{ox}}{d}=1.73\;\mathrm{fF},$$

*d* is the thickness of the SiO_2_ passivation layer on the source and drain contacts [19].

For this small value of *C_pd_*, peaks do not appear in the spectrum and the effect of *z*_1_ is negligible. Also, *p*_2_ gets shifted to higher frequencies beyond 1 GHz (not shown in the figure). The results clearly show the effect of the biomolecular layer on the transfer function of the SiNW-FET sensor. The change in the pole *p*_3_ and the zero *z*_2_ dominate the transfer function spectrum in the investigated frequency range (up to 100 MHz). It is worthwhile to mention that improvement in the visibility of *p*_3_ could be achieved by using high-κ dielectric material such as HfO_2_ instead of SiO_2_ for the gate oxide of the SiNW-FET sensor. Furthermore, the drain and source contact capacitances *C_ps_* and *C_pd_* need to be minimized so that effect of biomolecules that bind to the gate of the SiNW-FET become the major contribution in the recorded spectra.

## 6. Conclusions

In this paper, we employed a behavioral model for a SiNW-FET in SPICE for biomolecular sensing by extracting data from impedance spectra. Initially, we propose an *ac* small-signal model for the SiNW-FET and the mathematical expression for the transistor-transfer function without any biomolecule binding at the surface of the SiNW-FET. The TTF is exhaustive in the sense that it involves various parameters described in the SiNW-FET model and the parameters of the transimpedance amplifier. For negligibly small values of parasitic capacitance *C_pd_*, the spectrum is similar to that of a low-pass filter with two poles. However, a finitely large value of *C_pd_* results in the occurrence of a zero frequency in the spectrum, which manifests as peaks in the TTF spectrum. Variations in the spectrum by altering the different parameters of the FET model have been investigated through simulations and the proposed mathematical expression of the TTF was validated.

For biomolecular sensing, the biomolecules were modelled as a parallel *RC* network. These biomolecules would largely interact with the gate of the SiNW-FET but would also interact with the drain and source contacts. This phenomenon can be electrically modelled by placing three parallel *R_bio_C_bio_* networks in series with *C_ox_*, *C_pd,_* and *C_ps_*. With *C_pd_* and *C_ps_* being considerably large, it would result in variation and shifting of the peak in the TTF spectrum as well as shift in the other pole frequencies. However, by implementing a microfluidic channel over the SiNW-FET and confining the binding of biomolecules within the microfluidic area, the effect of the parasitic capacitances of the contact lines could be significantly reduced, and the interaction of the biomolecules with the gate of the SiNW-FET becomes the major effect.

## Figures and Tables

**Table 1:** SPICE parameters and values for model of SiNW-FET [19].

Parameter	Value
*Width*	370 nm
*Length*	10 μm
*t_si_*	50 nm
*t_ox_*	6 nm
*V_th_*_0_	−0.4 V
*U*_0_	115 cm^2^/Vs
*V_sat_*	80,000
*N_ch_*	1 × 10^17^ cm^−3^
*A*_0_	−0.55
*A*_2_	1.08

**Table 2:** Simulation parameters for SiNW-FET and readout operational amplifier.

Parameter	Value
$R_{fb}$	270 kΩ
$C_{fb}$	1 pF
$R_{sol}$	5 kΩ
$C_{ps}$	50 pF
$C_{pd}$	33 pF
pH	7.0

## Abbreviations

The following abbreviations are used in this manuscript:

*X**j*	Junction depth
*V*_*sat*_	Saturation velocity
*U*_0_	Mobility
*N*_*ch*_	Doping concentration near channel interface
*V*_*th*0_	Threshold voltage
*A*_0_	Bulk charge coefficient
*A*_2_	Second non-saturation factor
*C*_*pd*_	Parasitic capacitance at drain contact
C_*ps*_	Parasitic capacitance at source contact
*R*_*sol*_	Resistance of electrolyte
*t*_*ox*_	Oxide thickness
*C*_*ox*_	Oxide capacitance
*A*_*ox*_	Gate-oxide interface area
*ε*_*ox*_	Permittivity of oxide
*g*_*m*_	Transconductance parameter
*C*_*fb*_	Feedback capacitance
*R*_*fb*_	Feedback resistance
*C*_*bio*_	Capacitance due to biomolecules
*R*_*bio*_	Resistance due to biomolecules
*t*_*bio*_	Thickness of biomolecular layer
*κ*_*bio*_	Relative permittivity of biomolecules
*ε*_*bio*_	Permittivity of biomolecules
*γ*	Capacitance per unit area of biomolecules
*λ*	Resistance per unit area of biomolecules
*A*	Area of contact with source or drain
*d*	Thickness of passivation layer
